# SIRT1 Activation by Natural Phytochemicals: An Overview

**DOI:** 10.3389/fphar.2020.01225

**Published:** 2020-08-07

**Authors:** Concetta Iside, Marika Scafuro, Angela Nebbioso, Lucia Altucci

**Affiliations:** Department of Precision Medicine, University of Campania “Luigi Vanvitelli”, Naples, Italy

**Keywords:** sirtuin 1, natural compounds, oxidative stress, human disorders, polyphenols

## Abstract

Sirtuins are class III histone deacetylases, whose enzymatic activity is dependent on NAD^+^ as a cofactor. Sirtuins are reported to modulate numerous activities by controlling gene expression, DNA repair, metabolism, oxidative stress response, mitochondrial function, and biogenesis. Deregulation of their expression and/or action may lead to tissue-specific degenerative events involved in the development of several human pathologies, including cancer, neurodegeneration, and cardiovascular disease. The most studied member of this class of enzymes is sirtuin 1 (SIRT1), whose expression is associated with increasing insulin sensitivity. SIRT1 has been implicated in both tumorigenic and anticancer processes, and is reported to regulate essential metabolic pathways, suggesting that its activation might be beneficial against disorders of the metabolism. *Via* regulation of p53 deacetylation and modulation of autophagy, SIRT1 is implicated in cellular response to caloric restriction and lifespan extension. In recent years, scientific interest focusing on the identification of SIRT1 modulators has led to the discovery of novel small molecules targeting SIRT1 activity. This review will examine compounds of natural origin recently found to upregulate SIRT1 activity, such as polyphenolic products in fruits, vegetables, and plants including resveratrol, fisetin, quercetin, and curcumin. We will also discuss the potential therapeutic effects of these natural compounds in the prevention and treatment of human disorders, with particular emphasis on their metabolic impact.

## Introduction

Epigenetic modifications are associated with genome stability, gene transcription, and metabolic regulation. Acetylation is one of the most characterized histone modifications. Histone acetyltransferase (HAT) and histone deacetylase (HDAC) enzymes control the levels of histone acetylation, modulating gene expression (Cavalli and Heard, 2019).

Sirtuins (SIRT) 1–7 are enzymes classified as class III HDACs. They exhibit different subcellular localizations: SIRT1, SIRT6, and SIRT7 are nuclear (although SIRT1 isoforms were also identified in the cytoplasm); SIRT2 is mainly cytosolic; SIRT3, SIRT4, and SIRT5 are mitochondrial and can shuttle to the nucleus (Chang and Guarente, 2014).

The enzymatic activity of SIRTs is dependent on NAD^+^ as a cofactor and plays an important role in controlling gene expression, DNA repair, metabolism, oxidative stress response, mitochondrial function, and biogenesis. Deregulation of their activity may lead to tissue-specific degenerative events that underlie several human pathologies, including cancer, diabetes, and cardiovascular diseases (Haigis and Sinclair, 2010; O’Callaghan and Vassilopoulos, 2017; Waldman et al., 2018). The most studied member of this enzymatic class is SIRT1. SIRT1 regulates metabolic pathways, cell survival, cellular senescence, and inflammation, and acts in the pathogenesis of chronic conditions such as diabetes as well as pulmonary, neurodegenerative, and cardiovascular diseases. Indeed, SIRT1 has been reported to play a key role in tumorigenesis, as an oncogene or tumor suppressor depending on the context specificity (Biason-Lauber et al., 2013). It is able to control these processes *via* deacetylation of lysine groups of histone and non-histone proteins, including known transcription factors (FOXO, MyoD, p53, PGC-1α) (Kupis et al., 2016).

Chronic inflammation caused by oxidative damage increases the risk of many chronic disorders, including heart, cardiovascular, and neurodegenerative diseases, obesity, insulin resistance, and type 2 diabetes (T2D) (Geto et al., 2020). Oxidative stress plays a key role in the pathogenesis of these conditions. The overproduction of reactive oxygen species (ROS), including free radicals, and reactive nitrogen species (RNS) can lead to damage of cellular components, such as lipids, proteins, and DNA. The imbalance between oxidants and antioxidants can result in cellular dysfunction, apoptosis, and necrosis (Liguori et al., 2018).

SIRT1 guards against oxidative stress by activating gene transcription of PGC-1α *via* deacetylation, and by regulating transcription of factors such as the nuclear receptor peroxisome proliferator-activated receptor (PPAR), nuclear respiratory factor (NRF), and mitochondrial transcription factor A (TFAM), involved in modulation of biogenesis and mitochondrial function (Ren et al., 2019), and metabolism of glucose and lipids (Rodgers et al., 2005). SIRT1 is also able to regulate the expression of superoxide dismutase (SOD) and glutathione peroxidase (Sun et al., 2018). In addition, since mitochondrial dysfunction leads to the activation of apoptosis, SIRT1 can directly regulate the apoptotic process by modulating acetylation of PGC-1α (Zhang et al., 2019). SIRT1 also regulates inflammatory response (Kauppinen et al., 2013). By modulating the acetylation level of NF-κB p65, SIRT1 is able to control transcription of genes such as IL (interleukin)-1, tumor necrosis factor α (TNF-α), IL-8, IL-6, and other inflammatory factors (Rodgers et al., 2005; Ren et al., 2019; Yeung et al., 2019). Through NF-κB, SIRT1 also regulates the expression of genes such as inhibitor of apoptosis protein (IAP) and B-cell lymphoma-2 (Bcl-2) and tumor necrosis factor receptor (TNFR) (Ren et al., 2019).

SIRT1 protects against oxidative stress *via* regulation of FOXO protein acetylation, which is involved in antioxidant processes, apoptosis, and cell proliferation (Wong and Woodcock, 2009). By activating FOXO/MsSOD pathway, SIRT1 increases the expression of manganese superoxide dismutase (MnSOD) and catalase, counteracting oxidative stress and promoting damage repair (Gu et al., 2016). SIRT1 also increases the expression of MnSOD by deacetylating p53, thus enhancing cellular antioxidant capacity (Brunet et al., 2004; Zhang et al., 2017; Ren et al., 2019).

Over the past few years, the ever-growing awareness that good health goes hand in hand with a healthy and balanced diet has encouraged people to eat more fruit and vegetables, and to take supplements to make up for any deficiency (D’Angelo et al., 2019). Bioactive compounds in the diet can act as antioxidant and anti-inflammatory agents, thereby reducing the negative effects of oxidative stress and the incidence of chronic diseases such as obesity, diabetes, and cardiovascular disorders (Wang et al., 2014). Several molecules, including natural phytochemical compounds, can modulate SIRT1 activity (Miceli et al., 2014). Numerous studies have provided evidence of the protective effects of natural polyphenolic substances such as resveratrol, quercetin, curcumin, and fisetin, and of natural non-polyphenolic substances such as berberine (McCubrey et al., 2017). Natural polyphenols are the largest group of phytonutrients and are considered potential agents for the prevention and treatment of stress-related oxidative diseases. They are found in many plants and foods, such as fruits, vegetables, tea, cereals, and wine, and long-term intake is associated with health benefits. Mediterranean diets are in fact linked to a reduced risk of chronic diseases due to the consumption of olive oil and red wine, which contain high amounts of polyphenols (Romagnolo and Selmin, 2017).

**Figure 1:**
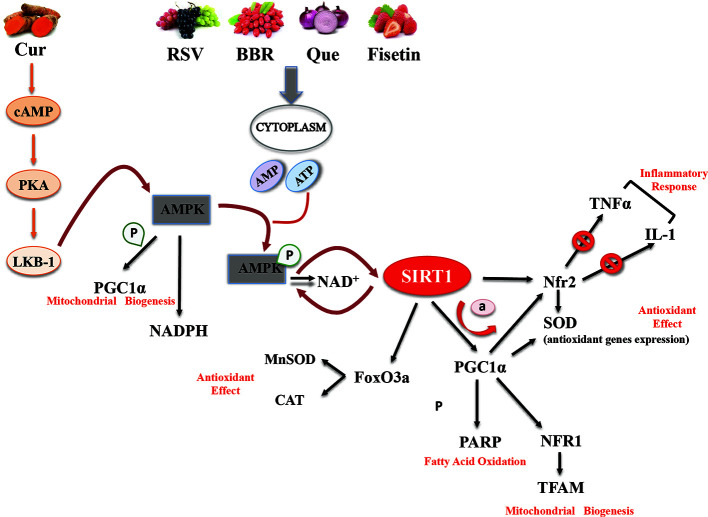
Basic mechanisms and effects of SIRT1 activation by polyphenol and non-polyphenol molecules.

Most of the evidence supporting the beneficial effects of phytochemical compounds comes from *in vitro* or animal studies, while human studies evaluating the long-term impact of phytomolecules are particularly few or inconsistent. Interventional studies are in fact limited by issues of bioavailability and metabolism. However, *in vitro* studies aimed at identifying cellular targets linked to the beneficial actions of phytonutrient-rich foods at concentrations ranging from nM to µM challenge the translatability of data. After ingestion, these compounds are in fact detected as phase II metabolites and their blood level does not exceed concentrations in the nM range. Substantial amounts of the compounds and their metabolites are degraded in the colon by intestinal microbiota, giving rise to small phenolic acids and aromatic catabolises which are absorbed by the circulatory system (Del Rio et al., 2013). Interesting studies showed that these natural polyphenol and non-polyphenol substances could affect SIRT1 expression/activity (**Table 1**) (de Boer et al., 2006). The main mechanisms of action common to polyphenol and non-polyphenol molecules that lead to antioxidant and anti-inflammatory effects *via* SIRT1 activation are reported in **Figure 1**.

**Table 1:** Classification of nutraceuticals based on their action and food source.

Natural SIRT1 activators	Effect	Source	References
Resveratrol	Positive effect on blood lipid profile, antioxidant	Dark grapes, raisins, peanuts	D’Angelo (2019) #44 Zordoky (2015) #54
Quercetin	Anticancer, positive effect on blood lipid profile, antioxidant, anti-inflammatory	Fruits, vegetables, nuts	Hung (2015) #72 Nabavi (2012) #68
Berberine	Antioxidant, anti-inflammatory	Natural component of traditional Chinese herb *Coptidis rhizoma*	Nabavi (2012) #68 Wu (2014) #70 Hung (2015) #72
Curcumin	Anticancer, antioxidant, anti-inflammatory	Active component in *Curcuma longa*	Zendedel (2018) #92
Fisetin	Anticancer, cardiovascular preventive, anti-inflammatory, antioxidant	Apples, persimmons, grapes, onions, kiwi, kale, strawberries	Kim (2015) #98 Chen et al., 2015)

**Figure 2:**
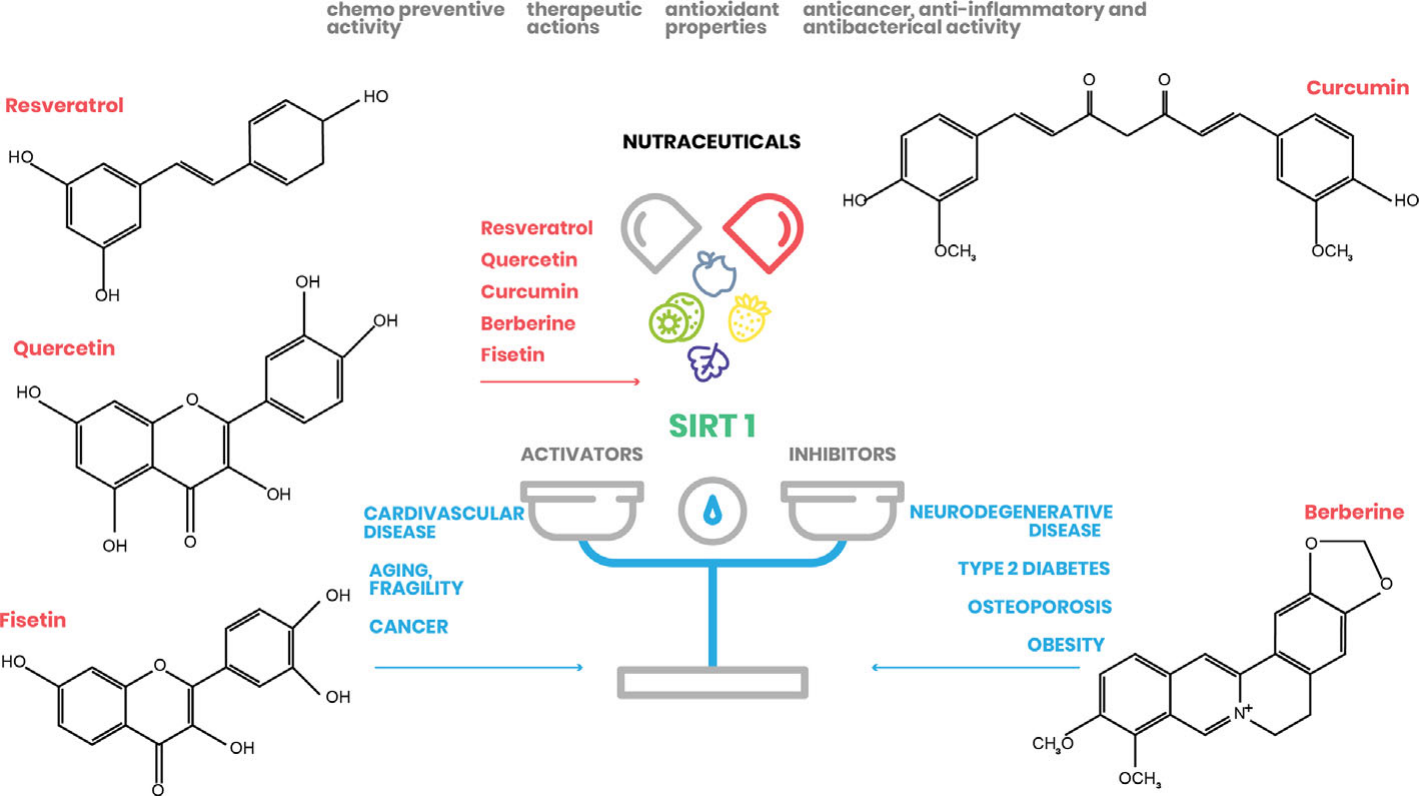
Nutraceutical action on SIRT1 expression. Natural substances have beneficial effects on human health by regulating SIRT1 action in different cellular processes (www.pubchem.ncbi.nlm.nih.gov).

Here, we focus on the natural molecules resveratrol, quercetin, fisetin, curcumin, and berberine, and elucidate their effect on SIRT1 activation and their potential to treat and/or prevent several human pathologies, mainly associated with metabolic disorders (**Figure 2**).

## Natural Compounds Enhancing SIRT1 Expression and Activity

### Resveratrol

Resveratrol (RSV), a non-flavonoid polyphenol found in grapes and grape products such as red wine, exerts an antioxidant action with reported cancer preventive properties (Kris-Etherton et al., 2002). RSV also has anti-inflammatory, anticancer, and anti-neurodegenerative effects (Piotrowska et al., 2012). The role of RSV as an immune response modulator was demonstrated in both *in vitro* and *in vivo* studies, where it reversed immune senescence in older rats, reduced inflammatory responses in rodents, and improved immunological activity against cancer cells (Malaguarnera, 2019). RSV was shown to be involved in the activation of macrophages, T cells, and natural killer cells, as well as in the suppression processes of CD4^+^ CD25^+^ regulatory T cells (Yang et al., 2008; Svajger and Jeras, 2012). All these effects are due to its ability to remove ROS, inhibit cyclooxygenase (COX), and trigger anti-inflammatory pathways *via* SIRT1 activation (Miceli et al., 2014; Malaguarnera, 2019). Activated SIRT1 interrupts TLR4/NF-κB/STAT axis, reduces cytokine production by inactivated immune cells, and inhibits pro-inflammatory factors derived from macrophages/mast cells, such as platelet-activating factor and TNF-α (Capiralla et al., 2012).

RSV-SIRT1 interaction modifies SIRT1 structure and promotes binding activity with its substrates including p65/RelA (Yeung et al., 2004), a component of the NF-κB complex, which regulates activation of leukocytes and inflammatory cytokines. SIRT1 activated by RSV inhibits acetylation of RelA by reducing the expression of inflammatory factors such as TNF-α, IL-1β, IL-6, metalloprotease (MMP)-1, MMP-3, and NF-κB-mediated Cox-2 (Malaguarnera, 2019). AMP activated protein kinase (AMPK) is also a target of RSV, as it controls SIRT1 activity *via* regulation of cellular levels of NAD^+^, thus acting as an energy sensor (Price et al., 2012). Cyclic adenosine monophosphate (cAMP) levels activate protein kinase A, resulting in phosphorylation and activation of SIRT1 (Wan et al., 2016). Activated SIRT1 catalyzes the deacetylation and activation of PGC-1α, thereby promoting beneficial effects in the metabolism (Ren et al., 2019).

In different organisms (*S. cerevisiae*, *C. elegans*, and *D. melanogaster*) expressing SIRT1 (or its homologous genes), RSV treatment is able to extend life span. In mammalians, RSV administration can improve SIRT1-dependent cellular processes such as axonal protection (Araki et al., 2004), fat mobilization (Chaplin et al., 2018), and inhibition of NF-κB-dependent transcription (Yeung et al., 2019); these effects are abolished in SIRT1 knockdown models. Numerous studies investigated the beneficial effects of RSV in cardiovascular diseases including hypertension (Theodotou et al., 2017), cardiac ischemia (Fourny et al., 2019), and atherosclerosis (Chassot et al., 2018). RSV has an effect on blood vessels, reduces inflammation, and prevents thrombus formation and platelet oxidation (Zordoky et al., 2015). It can also reduce cardiac dysfunction, oxidative stress, fibrosis, and apoptosis in the heart (Gupta et al., 2014; Yamagata, 2019). In addition, RSV was found to improve heart and kidney damage in rats (Li et al., 2020a). The protective effect of RSV is associated with an increase in SIRT1 activity, which deacetylates FOXO1 and activates MnSOD downstream. RSV-induced MnSOD also reduces oxidative stress (Li et al., 2020a). A recent *in vitro* study showed that RSV reduces hypoxia-induced apoptosis in H9C2 cells through activation of SIRT1/miR-30d-5p/NF-κB axis (Han et al., 2020). RSV treatment decreased cortical and hippocampal malondialdehyde levels while increasing SOD activity and SIRT1 expression in a diabetic rat model (Ma et al., 2019).

RSV was shown to activate SIRT1 and improve endothelial function in obese mice *via* upregulation of PPARδ expression/activity in PPARδ mutant mice (Cheang et al., 2019). It had previously been observed that Akt activation together with PPARδ is involved in vascularization of db/db mice (Tian et al., 2012); RSV was subsequently reported to increase phosphorylation of Akt and transcriptional activity of PPARδ in the aorta of wild-type mice, thus supporting the hypothesis of SIRT1-PPARδ interaction, and to strongly decrease LPC-induced mitochondrial ROS in the aortic endothelium of C57BL/6 mice (Cheang et al., 2019). Taken together, these findings highlight the beneficial effects of RSV against oxidative stress, which is involved in major pathologies such as heart and metabolic disorders. Although RSV is beneficial in many contexts, its pleiotropic actions need to be better studied in order to understand which of its described activities are directly due to SIRT1 modulation and whether this effect is always direct.

Because of the pleiotropic actions of RSV, clinical trials are currently testing its therapeutic potential in a wide range of human diseases. However, of all the mechanisms described in *in vitro* and *in vivo* studies, only a few have been confirmed in humans, such as gene and protein regulation in blood or muscle cells, and Akt signaling pathways (Ghanim et al., 2010; Brasnyo et al., 2011). Many clinical studies conducted in healthy patients and volunteers using both high and low doses of RSV highlight its potential cardioprotective benefit through improvement of endothelial function, inflammatory markers, and glucose metabolism. Nevertheless, the mechanisms of action are not yet well defined. Despite clinical evidence of its effects, the poor bioavailability and rapid metabolism of RSV severely limit the potential use of this molecule in the clinic. Future scientific research should focus on identifying actual metabolites or mediators of these observed effects.

To date, 165 clinical trials have tested the efficacy, safety, and pharmacokinetics of RSV in the prevention and treatment of 32 different pathological conditions (www.clinical.trials.gov). Restricting the search to interventional phase 1/2/3/4 studies (completed and terminated clinical trials), 55 addressed the ability of RSV to improve the pathological conditions of patients affected by several diseases. Most of these studies tested RSV-mediated effects in central nervous system disorders (Friedreich ataxia, Alzheimer’s disease, Parkinson’s disease), metabolic disorders [T2D, insulin resistance, dyslipidemia, hypercholesterolemia, metabolic syndrome X, non-alcoholic fatty liver disease (NAFLD)]. A phase 1 clinical trial (NCT01640197) tested the effects of chronic resveratrol supplementation (500 mg daily for 28 days) in healthy humans and found considerable improvements in cognitive performance, cerebral blood flow, subjective sleep, mood, health, and blood pressure. A list of completed and terminated clinical trials in which RSV was tested for metabolic disorders is reported in **Table 2**. Focusing on completed clinical trials with available results, NCT02114892 evaluated the effect of RSV on metabolic syndrome, demonstrating that, when administered three times per day (1,500 mg/die) before meals, RSV was able to treat and protect from obesity and diabetes with beneficial effects on glucose and lipid metabolism, blood pressure, and body weight. Another phase 1/2 study (NCT02095873) evaluated the effects of a formulation composed of RSV and hesperetin in obese subjects and found that these molecules are dietary inducers of glyxalase 1, improving metabolic and vascular health of obese subjects (Xue et al., 2016).

**Table 2:** Resveratrol in clinical trials for metabolic disorders.

Status	Study Title	Conditions	Intervention	Phase	NCT Number
Completed	Effects of Resveratrol in Patients With Type 2 Diabetes	Type 2 Diabetes	500 mg to a maximum dose of 3 g daily	Phase 1	NCT01677611
Terminated	Effect of Administration of Resveratrol on Glycemic Variability in Individuals With Type 2 Diabetes Mellitus	Type 2 Diabetes Mellitus	500 mg 3 times daily	Phase 2	NCT02549924
Completed	Effect of Resveratrol on Age-related Insulin Resistance and Inflammation in Humans	Type 2 Diabetes Mellitus & Insulin Resistance	1,000 mg twice daily for 28 days	Phase 2	NCT01354977
Completed	Regulation of Intestinal and Hepatic Lipoprotein Secretion by Resveratrol	Dyslipidaemia & Insulin Resistance	500 mg for 1 week followed by 1 g for 1 week	Phase 2	NCT01451918
Completed	Effects of Dietary Antioxidants to Prevent Cardiovascular Disease	Hypercholesterolemia & Healthy	Dietary Supplement: red wine for 1 month Dietary Supplement: resveratrol for 1 month	Phase 2	NCT02409537
Completed with results	Healthy Aging Through Functional Food	Glucose Intolerance & Aortic Stiffness & Vasodilation	Trans-resveratrol, 90 mg + hesperetin, 120 mg (combination)	Phase 1/2	NCT02095873
Completed	Effects of Resveratrol on Inflammation in Type 2 Diabetic Patients	Type 2 Diabetes Mellitus & Inflammation & Insulin Resistance & Other Disorders of Bone Density and Structure	6 months 40 mg daily then 6 months 500 mg daily	Phase 3	NCT02244879
Completed with results	Effect of Resveratrol Administration on Metabolic Syndrome, Insulin Sensitivity and Insulin Secretion	Metabolic Syndrome X	500 mg 3 times daily before meals with a total dose of 1,500 mg daily	Phase 2	NCT02114892
Completed	Resveratrol for the Treatment of Non Alcoholic Fatty Liver Disease and Insulin Resistance in Overweight Adolescents	NAFLD & Type 2 Diabetes & Metabolic Syndrome	75 mg twice daily for a total daily dose of 150 mg for 30 days	Phase 2/3	NCT02216552
Completed	A Study of Resveratrol as Treatment for Friedreich Ataxia	Friedreich Ataxia	1 g daily (500 mg twice daily) for 12 weeks then 5 g daily (2.5 g twice daily) for 12 weeks	Phase 1/2	NCT01339884
Completed	Effect of Banaba (Lagerstroemia Speciosa) on Metabolic Syndrome, Insulin Secretion and Insulin Sensitivity	Metabolic Syndrome X	Banaba capsules, 500 mg, 2 times daily before meals for 90 days	Phase 2	NCT02767869

### Quercetin

The flavonoid polyphenol quercetin (Que; 3,3′,4′,5,7-pentahydroxyflavone) is a natural, safe dietary supplement found in a glycoside form in fruits, vegetables, and nuts, which has antioxidant and anti-inflammatory properties (Nabavi et al., 2012; Wu et al., 2014).

In recent years, the scientific community has focused on the potential antiproliferative, chemopreventive and anticarcinogenic activities of Que as well as on its role as a modulator of gene expression. However, Que was also found to have potentially toxic effects including mutagenicity, pro-oxidant activity, mitochondrial toxicity, and inhibition of enzymes involved in hormonal metabolism (Li et al., 2016). Due to its poor solubility, short half-life, and low bioavailability, its medical use is limited (Konrad and Nieman, 2015). In humans, Que bioavailability is very low (~ 2%) and absorption varies from 3% to 17% in subjects receiving 100 mg/die (Costa et al., 2016). Que may reduce infection (Li et al., 2016), hepatic lipemic-oxidative damage (Cui et al., 2013) (Zhang et al., 2016b), and antioxidant risk (Xu et al., 2019). In addition, Que is known to exert a modulating action on immunity (Galleggiante et al., 2019). As regards its mechanism of action, in some cell lines Que was able to inhibit the production of TNF in macrophages (Tang et al., 2019), IL-8 in A549 lung cells induced by lipopolysaccharide (LPS) (Geraets et al., 2007), and TNF-α and IL-1α mRNA levels in glial cells, causing a decrease in neuronal cell death induced by microglial activation (Li et al., 2016). Mainly in immunity and inflammation, Que acts on leukocytes and targets many intracellular signaling kinases and phosphatases as well as enzymes and membrane proteins (Li et al., 2016). The immunostimulating effect of Que is due to induction of the expression of interferon-γ (IFN-γ) derived from Th-1 and inhibition of IL-4 derived from Th-2 in normal peripheral blood mononuclear cells (Nair et al., 2002). In addition, Que reduces T cell proliferation by blocking IL-12-induced tyrosine phosphorylation of JAK2, TYK2, STAT3, and STAT4 (Muthian and Bright, 2004; Nabavi et al., 2012). In inflammation, Que inhibits the enzymes COX and lipoxygenase (Lee and Min, 2013; Savikin et al., 2017). In the RAW 264.7 cell line, Que was also shown to counteract LPS-induced inflammation by phosphorylation of tyrosine phosphatidylinositol-3-kinase (PI3K)-p85 and complex formation of toll-like receptor 4 (TLR4)/MyD88/PI3K (Endale et al., 2013).

Oxidative stress occurs following an imbalance of the body’s antioxidant defence mechanisms and excessive generation of free radicals, and is involved in various pathologies such as diabetes, atherosclerosis, hypertension, neurodegenerative diseases, inflammation, and cancer (Oboh et al., 2016). Que is a powerful ROS scavenger and its antioxidant action is due to the presence of two pharmacophores within the molecular structure, which confer a favorable configuration for free radical elimination (Costa et al., 2016). Generally, Que reduces the effects of free radicals by transferring the hydrogen atom and stabilizing the radicals, a feature that has a structure-function relationship (Oboh et al., 2016).

Que can also act as both an antioxidant and pro-oxidant agent. At low concentrations (10–10 µM), Que displayed a protective effect against oxidative DNA damage (*in vitro*) in human lymphocytes (Li et al., 2016). At concentrations between 5 µM and 50 µM, Que was able to directly eliminate ROS *in vitro* (Costa et al., 2016). However, its effect *in vivo* is very likely not direct but due to its ability to modulate the cell’s antioxidant defense mechanisms; moderate oxidative stress can in fact increase the cell’s antioxidant defenses, resulting in general cytoprotection (Halliwell et al., 2005). Recent research showed that oxidized LDL (oxLDL) induces oxidative stress (Lara-Guzman et al., 2018). Oxidative injuries promote ROS generation in human endothelial cells and SIRT1 regulates endothelial function. Therefore, enhancement of SIRT1 activity and SIRT1/AMPK axis upregulation inhibits oxidative injury, inducing endothelial dysfunction (Chen et al., 2013) (Shentu et al., 2016). Que may reduce oxLDL-induced oxidative damage by upregulating SIRT1 and AMPK (Hung et al., 2015), therefore potentially preventing oxLDL-impaired SIRT1 inhibition linked to endothelial dysfunction. These findings indicate that SIRT1 can function as a regulator to improve AMPK activity under oxLDL stimulation (Hung et al., 2015).

It was very recently shown that Que (70 mg/kg) can reduce insulin resistance and improve glucose metabolism by reducing sensitivity to T2D/insulin resistance in ob/ob mice *via* SIRT1 activation (Hu et al., 2020). In this context, another study showed that in streptozotocin-induced diabetic rats, Que (100 mg/kg) inhibits oxidative damage by increasing SIRT1 expression and decreasing levels of NF-κB, a SIRT1 substrate (Iskender et al., 2017).

In recent years, the scientific community has focused on the role of apoptosis in cardiovascular disease, showing that oxidative stress, myocardial ischemia, hypoxia, and ischemia/reperfusion injury may induce myocardial apoptosis (Donniacuo et al., 2019). Tang and colleagues evaluated the effects of Que in improving myocardial ischemia/reperfusion injury (MI/R)-induced cell apoptosis both *in vitro* and *in vivo*. SIRT1 and PGC-1α expression levels were decreased in rat MI/R groups, but were significantly increased after treatment with Que (Tang et al., 2019). Furthermore, activation of SIRT1/PGC-1α pathway upregulated Bcl-2 expression and downregulated Bax, exerting anti-apoptotic effects. The authors hypothesized that Que might improve MI/R-induced myocardial damage *via* regulation of SIRT1/PGC-1α and Bcl-2/Bax pathways (Tang et al., 2019). Que is also reported to regulate ROS generation and mitigate mitochondrial dysfunction by promoting their biogenesis. Specifically, in a study to develop a therapeutic strategy for osteoarthritis, Que was shown to increase expression levels of SIRT1, PGC-1α, NRF1 and NFR2, TFAM, and phospho-AMPK α in osteoarthritis rats, confirming the hypothesis that Que might act *via* the AMPK/SIRT1 signaling pathway (Qiu et al., 2018). Overall, these findings suggest that Que may counteract cardiovascular disease and oxidative damage.

The growing body of evidence supporting the beneficial effects of Que has led to its clinical use, as demonstrated by the number of clinical trials (72 studies on ClinicalTrials.gov). A list of completed studies using Que in different metabolic and inflammatory conditions is reported in **Table 3**. Specifically, a phase 2 clinical trial (NCT01839344) measured the effect of Que on glucose tolerance and postprandial endothelial function in subjects with T2D compared to the effect of an alpha-glusidase inhibitor, acarbose. The administration of 2 g of Que led to a decrease in postprandial blood glucose (NCT01839344). Given its antioxidative and anti-inflammatory capacities, this flavonoid was considered a good candidate for antioxidant therapy in mucositis (NCT01732393), hepatitis C (NCT01438320), idiopathic pulmonary fibrosis (NCT02874989), osteoporosis (NCT00330096), uric acid metabolism (NCT01881919), cytokine release (NCT01106170), and chronic obstructive pulmonary disease (NCT01708278). In the latter study, Que supplementation was safely tolerated by patients with mild-to-severe chronic obstructive pulmonary disease, opening the way towards the potential use of Que as a therapeutic agent for this condition.

**Table 3:** Quercetin in clinical trials for metabolic and inflammatory disorders.

Status	Study Title	Conditions	Intervention	Phase	NCT Number
Completed	Effect of Quercetin in Prevention and Treatment of Oral Mucositis	Chemotherapy Induced Oral Mucositis	250 mg daily for 3 weeks	Phase 1/2	NCT01732393
Completed with results	Beneficial Effects of Quercetin in Chronic Obstructive Pulmonary Disease (COPD)	Chronic Obstructive Pulmonary Disease	500 to 2,000 mg daily for 1 week	Phase 1	NCT01708278
Completed	Q-Trial in Patients With Hepatitis C	Chronic Hepatitis C	28 days	Phase 1	NCT01438320
Completed	Effects of Quercetin on Blood Sugar and Blood Vessel Function in Type 2 Diabetes.	Diabetes Mellitus, Type 2	250 mg; oral single dose of 2000 mg	Phase 2	NCT01839344
Completed	Effect of Quercetin Supplements on Healthy Males: a 4-Week Randomized Cross-Over Trial	Hyperuricemia, Gout, Kidney Calculi, Diabetes, Cardiovascular Disease	500 mg tablet for 28 days with meal (breakfast preferred.)	Early Phase 1	NCT01881919
Completed	Targeting Pro-Inflammatory Cells in Idiopathic Pulmonary Fibrosis: a Human Trial	Idiopathic Pulmonary Fibrosis (IPF)	3 doses administered over 3 consecutive days in 3 consecutive weeks, oral administration of quercetin (1,250 mg daily)	Phase 1	NCT02874989
Completed	Efficacy of Provex CV Supplement to Reduce Inflammation Cytokines and Blood Pressure	Blood Pressure	330 mg of Provex CV supplement, by mouth, per day, for 4 weeks	Phase 1	NCT01106170
Completed	Effects of Hesperidin on Bone Mineral Density and Bone Metabolism of Postmenopausal Women	Osteoporosis, Osteopenia		Phase 3	NCT00330096

However, as for RSV and nutraceuticals in general, the results of molecular studies on Que obtained from *in vitro* investigations and animal models are often inconsistent with data from clinical trials. Concentration factor (dose and timing of administration) and bioavailability are the two main issues that require further clarification. Additional studies are needed to identify the optimal concentration of Que for it to exert a beneficial effect, for example on insulin sensitivity.

### Berberine

Berberine (BBR) is an isoquinoline alkaloid reported to have analgesic, anticancer, anti-inflammatory, and myocardial protective properties (Cicero and Baggioni, 2016). It was found to exert protective antioxidative effects in different physiologic and pathologic conditions (Huang et al., 2018; Li et al., 2020b). However, the mechanisms underlying these effects remain unclear. BBR was described as a potential antitumor agent that induces cell cycle arrest in G0/G1 phase, increases Cip/p21 and Kip/p27 protein expression, decreases expression of cyclin D1, D2, and DE, and the cyclin-dependent kinases Cdk2, Cdk4, and Cdk6, promoting apoptosis in HL-60 human leukemia cells (Li et al., 2017). BBR can also deregulate telomerase activity and promote mitochondria-dependent apoptosis in HepG2 human hepatocarcinoma cells through caspase 8 and caspase 3 activation, PARP cleavage induction, increased expression of the pro-apoptotic protein Bax through activation of FOXO transcription factors, and inhibition of Bcl-2 and Bcl-x anti-apoptotic protein expression (Hwang et al., 2006). BBR was observed to exert an apoptotic effect by inducing ROS production and increasing MAPK and JNK activity of p38 in SW620 human colon carcinoma cells, and by increasing Ca^+^ and cytochrome C release in HSC-3 squamous cells (Song et al., 2020). In addition, BBR inhibits the proliferation of cancer cells through an anti-inflammatory pathway. In oral carcinoma cell lines and in SCC-4 cells, BBR inhibits expression of COX2 and AP-1 bond, decreases prostaglandin E2 (PGE2) production, and suppresses NF-κB, IKK, ERK, and JNK activities. Furthermore, BBR can inhibit colon cancer cell growth by activating retinoid X receptor α (RXRα), which binds RXRα, and promoting β-catenin degradation (Ruan et al., 2017). However, some studies highlighted the potential ability of BBR to prevent oxidative stress-induced senescence by activating AMPK and restoring NAD^+^ levels (Song et al., 2020).

Initial research revealed a significant role of SIRT1 signaling in mediating the antioxidant effect of BBR in diabetes (Pang et al., 2015) and in lipid metabolism (Hasanein et al., 2017). The lipid-lowering activity mediated by co-treatment with BBR and RSV was investigated in mice exposed to a high fat diet (Zhu et al., 2018). *In vivo* data showed that BBR combined with RSV lowered total cholesterol, triglyceride, and LDL cholesterol levels in mice. These findings were also confirmed *in vitro* with 3T3-L1 adipocytes treated with BBR or RSV alone. Specifically, BBR and RSV co-treatment was able to reduce lipid accumulation more robustly than single treatments. BBR in combination with RSV displayed hypolipidemic effects likely mediated by SIRT1 expression regulation. Moreover, BBR pre-treatment seemed to counteract SIRT1 downregulation (Zhu et al., 2018).

The antioxidant and anti-inflammatory effects of BBR were also investigated in heart (Yu et al., 2016). BBR-mediated SIRT1 activation reduced MI/R injury by affecting oxidative damage and inflammation signaling. Specifically, BBR exerted an antioxidant effect by decreasing the generation of cardiac superoxide and gp91^phox^ expression, and by increasing SOD levels (Yu et al., 2016). A previous study had also shown that SIRT1 activation promotes antioxidant molecule production and decreases pro-apoptotic proteins through FOXO1 activation, thus protecting against MI/R lesions (Hsu et al., 2010).

As well as activating SIRT1, BBR is also able to decrease FOXO1 acetylation, triggering anti-apoptotic signaling pathways *via* Bcl-2 expression, and Bax and caspase-3 downregulation (Yu et al., 2016).

A very recent report described the protective effect of BBR against doxorubicin-induced cardiovascular damage (Wu et al., 2019). This effect is mediated by SIRT1/p66Shc pathway (Sampaio et al., 2016). Data obtained in a rat model and in rat H9c2 cardiac cells showed that BBR treatment leads to upregulation of SIRT1 and downregulation of p66shc expression both *in vivo* and *in vitro*, resulting in suppression of ROS production, apoptosis, and mitochondrial damage, and improving cardiac dysfunction. This effect did not occur if H9c2 cells were treated with the SIRT1 inhibitor EX-527 (Bai et al., 2018), indicating that BBR action was dependent on SIRT1 (Wu et al., 2019). Another study investigated the beneficial effect of BBR in an *in vivo* diabetic mouse model and *in vitro* pancreatic beta cell NIT-1 high glucose treated to induced diabetic condition. BBR attenuated oxidative stress by increasing SOD1 levels through activation of SIRT1 and inhibition of miR-106b expression (Chen and Yang, 2017).

These promising results have paved the way towards the clinical use of BBR. A list of clinical studies investigating the beneficial effects of BBR in inflammatory and metabolic diseases is reported in **Table 4**. Given the experimental efficacy of the antidiabetic and antidyslipidemia action of BBR, most of the 18 completed or terminated clinical trials focused on these properties. Two clinical trials tested the anti-diabetic effect of BBR in patients with diabetes or diabetes and dyslipidemia (NCT00425009 and NCT00462046). Several clinical studies investigated its effects on hyperlipidemia and hypercholesterolemia and relative disorders. In particular, two phase 4 clinical trials (NCT02422927 and NCT03470376) tested the ability of a nutraceutical combination containing 500 mg BBR to ameliorate inflammation lipid profile and endothelial injury markers in patients with elevated levels of high-sensitivity C-reactive protein and in HIV-infected patients receiving stable antiretroviral therapy. In addition, a phase 2 clinical study (NCT03216811) assessed the efficacy of a nutraceutical compound (3 mg containing fermented red rice) in terms of cholesterol, and endothelial and inflammatory parameters in subjects with hypercholesterolemia and low-to-moderate cardiovascular risk. The findings of a very recent study suggest that BBR may be effective and safe to reduce cardiovascular risk associated with metabolic syndrome (Mercurio et al., 2020).

**Table 4:** Berberine in clinical trials for metabolic and inflammatory disorders.

Status	Study Title	Conditions	Intervention	Phase	NCT Number
Completed	Therapeutic Effects of Berberine in Patients With Type 2 Diabetes	Type 2 Diabetes		Phase 1/2	NCT00425009
Completed	Efficacy and Safety of Berberine in the Treatment of Diabetes With Dyslipidemia	Diabetes Mellitus, Type 2, Metabolic Syndrome	1 g daily	Phase 3	NCT00462046
Terminated	The Therapeutic Effects of Statins and Berberine on the Hyperlipemia	Dyslipidemias	500 mg twice daily for 8 weeks	Phase 4	NCT01697735
Completed	Efficacy and Tolerability of the Nutraceutical Formulation Coleosoma in Dyslipidemic Subjects (Coleosoma)	Dyslipidemias	Coleosoma 500 mg daily for 12 weeks	Phase 2	NCT03027336
Completed	Combined Effects of Bioactive Compounds in Lipid Profile (ARM-PLUS-LDL)	Hyperlipidemia, Low-density-lipoprotein-type, Elevated Triglycerides	One tablet per day during 12 weeks	Phase 2/3	NCT01562080
Completed	Long Term Efficacy and Tolerability of a Nutraceutical Combination (Red Yeast Rice, Policosanols and Berberine) in Low-moderate Risk Hypercholesterolemic Patients: a Double-blind, Placebo Controlled Study	Hypercholesterolemia	500 mg daily for 24 weeks	Phase 4	NCT02078167
Completed	NUtraceutical TReatment for hYpercholesterolemia in HIV-infected Patients (NU-TRY(HIV))	Hypercholesterolemia Inflammation Atherosclerosis	500 mg daily for 3 months	Phase 4	NCT03470376
Completed	Nutraceutical Combination in Patients With Low-grade Systemic Inflammation	Hypercholesterolemia Inflammation Atherosclerosis	500 mg for 3 months	Phase 4	NCT02422927
Completed	Nutraceutical in Cardiovascular Primary Prevention (NIRVANA)	Hypercholesterolemia	8-week administration of nutraceutical compound 3 mg	Phase 2	NCT03216811

### Curcumin

Curcumin (Cur) or 1,7-bis(4-hydroxy-3-methoxyphenyl)-1,6-heptadiene-3,5-dione (diferuloylmethane) is a natural bioactive polyphenol compound mediating a wide spectrum of biological functions. The molecular structure of Cur consists of two aromatic rings containing phenolic O-methoxy groups linked by a carbon bond, which has a β-unsaturated β-diketone fraction (Priyadarsini, 2014). Thousands of years ago, Indians and Chinese described the health-giving effects of this substance, derived from the turmeric plant (Gupta et al., 2014). Today, Cur is considered a potential agent with antioxidant, anticancer, and anti-inflammatory properties (Yu et al., 2019).

As an antioxidant, Cur is able to eliminate ROS and RNS by increasing the expression of antioxidant proteins *via* induction of upstream coding genes such as nuclear factor erythroid 2-related factor 2 (Nrf2), kelch-like ECH-associated protein 1 (Keap1), and antioxidant response element (ARE) (Abdelsamia et al., 2019). Nrf2 is a transcription factor involved in cellular stress response. Under normal conditions, the cystine-rich zinc Keap1 metalloprotein binds Nrf2 inside the cytoplasm, promoting ubiquitination and consequent proteasomal degradation and preventing Nrf2 nuclear translocation (Serafini et al., 2019). In contrast, under stress conditions Keap1 activity is inhibited by phosphorylation and free Nrf2 moves to the nucleus, where it binds to ARE in the regulatory regions of cytoprotective proteins and promotes transcription of antioxidant genes such as SOD, glutathione peroxidase (GPx), and GSH, and of phase II detoxifying enzymes such as heme oxygenase-1 (HO-1), glutathione transferases (GST), nicotinamide adenine dinucleotide phosphate (NADPH), and NAD(P)H dehydrogenase [quinone] 1 (NQO1) (Serafini et al., 2019). Cur is able to activate Nrf2/Keap1/ARE signaling pathway by Michael reaction with thiol residues in Keap1, inducing the release and activation of Nrf2 and promoting antioxidant effects (Wafi et al., 2019; Yu et al., 2019).

Cur was also shown to exert immunomodulatory activity by interacting with elements involved in inflammatory response including JAK/STAT pathway, suppressor of cytokine signaling (SOCS) expression, and TLR4/MyD88/NF-κB axis. Cur inhibits phosphorylation of JAK/STAT by binding with its α,β-unsaturated carbonyl portion to residue 259 of cysteine in STAT3 with subsequent activation. It is known that *in vitro*, even at 7.5 µM concentration, Cur is able to phosphorylate STAT3 and attenuate inflammatory response (Serafini et al., 2019). SOCS is an antagonist of JAK/STAT signaling and is involved in the regulation of inflammatory proteins and cytokines. *In vitro*, Cur restores expression of SOCS1 and SOCS3, and inhibits expression of IL-6, TNF-α, and PGE2 in RAW 264.7 macrophages (Guimaraes et al., 2013), while *in vivo* it restores immunological balance by acting on JAK/STAT/SOCS signaling, inhibiting JAK2, STAT3, and STAT6 phosphorylation and increasing SOCS1, SOCS3, and PIAS3 expression (Zhang et al., 2016a). Furthermore, some *in vitro* and *in vivo* studies show that Cur can downregulate TLR4, MyD88, and NF-κB signaling at neuronal level by inducing the release of pro-inflammatory cytokines (TNF-α, IL-1β, IL-6), producing an anti-inflammatory effect.

Cur is able to act on apoptosis and on mitochondrial biogenesis and dysfunction through SIRT activation by small molecules (Gupta et al., 2012). Cur can downregulate expression of growth factor receptors, transcription factors, TNFs, and nitric oxide synthase, and increase AMPK levels. In vascular smooth muscle cells, Cur promotes AMPK activation, which enhances superoxide and ATP production. This event stimulates an increase in NAD^+^ levels and SIRT1 activation (Zendedel et al., 2018). Cur-induced SIRT1 upregulation has beneficial effects against a range of diseases including cardiac fibrosis, diabetes, and ischemia/reperfusion injury (Zendedel et al., 2018 #7; Yang et al., 2013 #5). The involvement of mitochondrial oxidative damage in several diseases such as MI/R is well known (Yang et al., 2013). Yang and colleagues investigated SIRT1 action after Cur treatment and the manner in which Cur might attenuate MI/R-induced mitochondrial oxidative damage. *In vivo* and *in vitro* experiments on rat heart and cardiomyocytes showed that Cur pre-treatment had protective effects, decreasing myocardial infarct size (Yang et al., 2013). Heart injury and mitochondrial oxidative damage are characterized by ROS overproduction and a reduction in succinate dehydrogenase or COX activity, leading to mitochondrial respiratory chain deficiency (Yang et al., 2013). Cardiomyocytes treated by Cur displayed upregulation of SIRT1, COX, and succinate dehy-drogenase (SDH), and downregulation of Bax. These effects were abolished when cardiomyocytes were treated with SIRT1 siRNA. In these conditions, the levels of decreased SIRT1 abolished the effects of Cur treatment. These findings show that Cur-induced cardio-protection was mediated by SIRT1 (Yang et al., 2013). T2D is a chronic illness requiring continuing care to prevent long-term and acute complications. Cur may have positive effects in T2D by mediating the reduction of NF-κB expression in inflammatory pathway (Zendedel et al., 2018). NF-κB deregulation can influence other mechanisms in which SIRT1 and AMPK are involved, such as glucose absorption in skeletal muscle. Data obtained in diabetic mice models showed that supplementation of Cur for 8 weeks might promote indirect activation of SIRT1 through AMPK (Jimenez-Flores et al., 2014).

Over time, preclinical studies have shown that Cur can act in various human diseases including immunodeficiency, virus infections, rheumatoid arthritis, myocardial infarction, atherosclerosis, and diabetes (Hsu and Cheng, 2007). A phase I study was published for the first time in 2001. Patients were subjected to five different Cur doses (500, 1,000, 2,000, 4,000, and 8,000 mg) every day for 3 months, and data showed that Cur treatment up to 8,000 mg/day is not toxic (Hsu and Cheng, 2007). Another study investigated Cur-mediated effects in patients with chronic non-alcoholic pancreatitis, showing that patients had a strong reversal of lipid peroxidation (Durgaprasad et al., 2005). The development of new routes of administration and new formulations of Cur with better bioavailability could be fundamental for future therapeutic strategies.

A list of completed and terminated clinical trials investigating the effects of Cur in metabolic and inflammatory diseases is reported in **Table 5**. A phase 2 clinical study (NCT01925547) investigated the effect of Cur micelles on inflammation and lipid metabolism markers in subjects at risk for metabolic syndrome. Two other trials, a phase 2 (NCT02017353) and a phase 3 (NCT02099890) study, investigated the effect of Cur on inflammation induced by endometrial carcinoma and spinal cord injury, respectively. Interesting results from three phase 2/3 clinical studies showed that Cur (as capsules or gel) prevents or reduces radiation-induced dermatitis in breast cancer patients receiving radiotherapy, enhancing the function of normal tissues (NCT01246973, NCT01042938, NCT02556632).

**Table 5:** Curcumin in clinical trials for metabolic and inflammatory disorders.

Status	Study Title	Conditions	Intervention	Phase	NCT Number
Completed	Micellar Curcumin and Metabolic Syndrome Biomarkers	Metabolic Syndrome, Protection Against	20 mg of curcumin at the beginning, after 3 and 6 weeks	Phase 2	NCT01925547
Completed	Effect of Curcumin Addition to Standard Treatment on Tumour-induced Inflammation in Endometrial Carcinoma	Endometrial Carcinoma	2 g daily for 2 weeks	Phase 2	NCT02017353
Completed	The Effect of Diet on Chronic Inflammation and Related Disorders Following Spinal Cord Injury	Neuropathic Pain, Depression, Cognitive Impairment	400 mg Baseline/3 months/6 months	Phase 3	NCT02099890
Completed with results	Oral Curcumin for Radiation Dermatitis	Radiation-induced Dermatitis, Breast cancer	2 g 3 times daily for 1 week	Phase 2/3	NCT01246973
Completed with results	Curcumin for the Prevention of Radiation-induced Dermatitis in Breast Cancer Patients		2 g 3 times daily (~4–7 weeks)	Phase 2	NCT01042938
Completed with results	Prophylactic Topical Agents in Reducing Radiation-Induced Dermatitis in Patients With Non-inflammatory Breast Cancer (Curcumin-II)	Breast Carcinoma, Breast Carcinoma, Pain, Radiation-Induced Dermatitis,		Phase 2	NCT02556632

### Fisetin

The dietary flavonoid fisetin (3,3′,4,7-tetra-hydroxyflavone) is a natural polyphenol present in plants and fruits such as apples, persimmons, grapes, onions, kiwi, kale, and strawberries, whose daily intake is estimated to be about 0.4 mg (Khan et al., 2013). This human diet constituent is reported to exert some beneficial anticancer, cardiovascular preventive, anti-inflammatory, and antioxidant effects that support normal cell homeostasis and cytoprotection (Chen et al., 2015; Kim et al., 2015). Fisetin was shown to have neuroprotective activity in various Huntington’s disease models through ERK activation and to inhibit melanoma growth by suppressing Akt/mTOR1 pathway (Sechi et al., 2018). A recent study reported that fisetin reduces myocardial tissue damage in a reperfusion ischemia model by suppressing mitochondrial oxidative stress and inhibiting glycogen synthase kinase 3β (Shanmugam et al., 2018). As a polyphenol, fisetin can counteract oxidative stress and mediate immune response *via* AMPK/SIRT1 and Nfr2 pathways (see **Figure 1**). Fisetin was shown to increase SIRT1 expression and enhance SIRT1-mediated PPAR and FOXO1 deacetylation in 3T3L1 cells (Kim et al., 2015). Specifically, fisetin enhanced the association between SIRT1 and the PPARγ promoter, leading to a block of its transcriptional activity, adipogenesis, and lipid accumulation (Kim et al., 2015). Lipid accumulation is a common feature in NAFLD (Liou et al., 2018). Increased fatty acid synthesis and hepatocyte accumulation is associated with metabolic disorder (Liou et al., 2018). Fisetin treatment in NAFLD mice reduced epididymal adipose tissue, hepatocyte steatosis, and fatty acid synthesis. The data reported by Liou et al. showed an increase in AMPKα phosphorylation and SIRT1 expression levels, while *in vitro* results revealed a reduction in lipid accumulation and an increase in lipolysis. The beneficial effects of fisetin were also linked to its action as a caloric restriction mimetic (CRM) (Singh et al., 2018). Natural compounds such as metformin and resveratrol display the ability to mimic the effects of caloric restriction, acting on stress response and metabolic pathways (Ingram et al., 2006). A recent study investigated the neuroprotective effects of fisetin as a possible CRM against apoptosis, oxidative stress, aging, and neurodegeneration (Singh et al., 2018). The authors showed that aging of rat brain induced an increase in pro-inflammatory cytokines and that treatment with CRMs such as metformin can reduce inflammation (Singh et al., 2019). In an attempt to demonstrate that fisetin could have beneficial effects against neuro-inflammation comparable to metformin, Singh et al. showed that fisetin mediated protective effects *via* SIRT1 activation, enhancing NF-κB deacetylation and promoting inhibition of pro-inflammatory gene expression (Singh et al., 2019). Data suggested that fisetin may be a suitable therapeutic candidate for aging and neurological diseases.

However, there are only six currently available clinical trials using fisetin, most of which are still recruiting (**Table 6**). These pilot studies aim to test the anti-inflammatory efficacy of fisetin in Frail Elderly Syndrome (NCT03675724 and NCT03430037) and in symptomatic knee osteoarthritis patients (NCT04210986). Another phase 2 clinical study (NCT03325322) intends to evaluate the effects of oral fisetin on adipose tissue-derived mesenchymal stem/stromal cell function, kidney function, inflammation, and physical function in subjects with chronic kidney diseases and diabetes.

**Table 6:** Fisetin in clinical trials for inflammatory disorders.

Status	Study Title	Conditions	Intervention	Phase	NCT Number
Recruiting	Alleviation by Fisetin of Frailty, Inflammation, and Related Measures in Older Adults	Frail Elderly Syndrome	20 mg/kg daily orally for 2 consecutive days	Phase 2	NCT03675724
Recruiting	Alleviation by Fisetin of Frailty, Inflammation, and Related Measures in Older Women	Frail Elderly Syndrome	20 mg/kg daily orally for 2 consecutive days, for 2 consecutive months.	Phase 2	NCT03430037
Recruiting	Senolytic Drugs Attenuate Osteoarthritis-Related Articular Cartilage Degeneration: A Clinical Trial	Osteoarthritis, Knee	20 mg/kg for 2 consecutive days, followed by 28 days off, then 2 more consecutive days	Phase 1/2	NCT04210986
Recruiting	Inflammation and Stem Cells in Diabetic and Chronic Kidney Disease	Chronic Kidney Diseases, Diabetes Mellitus, Diabetic Nephropathies	20 mg/kg daily orally for 2 consecutive days	Phase 2	NCT03325322

## Conclusions

*In vitro* and *in vivo* studies as well as clinical trials in humans show that SIRT-activating compounds derived from natural sources could preserve human health and might prove beneficial for the prevention and treatment of a plethora of human diseases. The fact that many of these natural molecules are introduced through diet underscores the importance of dietary intervention to correct predisposition and life-style disorders. However, it remains unclear whether (or not) the effects of these compounds are mostly related to SIRT activation and what drug dose/concentration is required. Since most of the natural compounds described here exhibit pleiotropic effects, determining a direct link between SIRT activation and improvement in human health is very challenging. It is also evident that a more robust correlation between health effects and administration of the bioactive compounds needs to be established in order to understand their biological impact and their direct association with SIRT activation. An additional issue is that, although numerous studies have been carried out, the majority only address the role of SIRT1 and its pharmacological regulation. Our current knowledge of the potential pharmacological activation of the expression/activity of other SIRTs is largely incomplete. A greater insight into the selectivity and specificity of natural SIRT activators may help understand the myriad beneficial effects described to date. A further question is related to the cell/context-specific expression of some SIRTs, cofactor availability, and context-specific action of some of the described modulators. Further investigations will be required to provide a more detailed understanding. In addition, some natural SIRT modulators, such as Cur, display poor bioavailability and solubility. Treatment with higher bioavailable preparations of Cur derivatives may result in increased SIRT1-activating action, further substantiating the link between SIRT1, Cur, and therapeutic effects. The fact that studies indicate that the majority of SIRT activators (or at least SIRT1-targeting activators) exert both direct activating effects and indirect effects *via* modulation of SIRT1 downstream pathways complicates the interpretation of results and, particularly, the mining of data specifically dependent on direct SIRT1 binding and activation. Similarly, the activating effects resulting from SIRT1 binding are reported to be either dependent on the catalytic domain or related to different domains, leaving considerable uncertainty as to the activating binding mode and its context specificity upon drug response. Despite the generally encouraging data from *in vitro* and *in vivo* studies, supporting molecular evidence providing clues to these unanswered questions is still lacking. A better understanding of the molecular mechanisms of these natural molecules (or their derivatives) may lead to further and more focused development of their preclinical and clinical use.

## Author Contributions

CI, MS, AN, and LA contributed to the redaction of the manuscript and gave final approval of the manuscript.

## Funding

Authors were supported by VALERE: Vanvitelli per la Ricerca Program, MIUR20152TE5PK, EPICHEMBIO CM1406, the Campania Regional Government Lotta alle Patologie Oncologiche iCURE (CUP B21c17000030007), Campania Regional Government FASE 2:IDEAL (CUP B63D18000560007), MIUR Proof of Concept (POC01_00043), Vanvitelli per la Ricerca Program “AdipCare” (ID 263).

## Conflict of Interest

The authors declare that the research was conducted in the absence of any commercial or financial relationships that could be construed as a potential conflict of interest.

## Abbreviations

SIRT1, Sirtuin 1; HATs, Histone acetyl transferases; HDACs, Histone deacetylases; ROS, Reactive oxygen species; PPAR, Receptor peroxisome proliferator-activated receptor; NRF, Nuclear respiratory factor; TFAM, Mitochondrial transcription factor A; SOD, Superoxide dismutase; TNF-α, Tumor necrosis factor α; IAP, Apoptosis protein inhibitor; Bcl-2, B-cell lymphoma-2 family; MnSOD, Manganese superoxide dismutase; RSV, Resveratrol; Que, Quercetin; oxLDL, Oxidized LDL; BBR, Berberine; Cur, Curcumin; COX, Cytochrome c oxidase; T2D, Type II diabetes; NAFLD, Non-alcoholic fatty liver disease; CRM, Caloric restriction mimetic.
